# Central Retinal Artery Occlusion Following Cosmetic Blepharoplasty in a Young Patient: A Case Report

**DOI:** 10.1155/crop/5972407

**Published:** 2026-07-22

**Authors:** Abdul Hadi Hassan Mallick, Manahil Malik, Fatima Zahra, Haji Ismail, Haroon Tayyab

**Affiliations:** ^1^ Department of Ophthalmology and Visual Sciences, Aga Khan University, Karachi, Pakistan, aku.edu

**Keywords:** blepharoplasty complications, central retinal artery occlusion, periocular surgery, retinal ischemia, visual loss

## Abstract

**Purpose:**

The purpose of this study is to report a case of unilateral central retinal artery occlusion (CRAO) as a rare ophthalmic complication following bilateral cosmetic blepharoplasty in a young patient and to highlight its probable causes, management, and preventive considerations.

**Methods:**

A 31‐year‐old gentleman, with no known comorbidities, presented with complaints of vision loss in the left eye for 6 weeks after undergoing bilateral cosmetic blepharoplasty. At presentation, the best corrected visual acuity (BCVA) was 20/25 in the right eye (OD) and no perception of light (NPL) in the left eye (OS). Intraocular pressure (IOP) was 14 mmHg in both eyes (OU). Pupillary examination revealed a round, regular, and reactive pupil in the right eye, whereas the left pupil was mid‐dilated and nonreactive, with a positive relative afferent pupillary defect (RAPD). The fellow eye remained asymptomatic following surgery.

**Results:**

At presentation, optical coherence tomography (OCT) was performed, and it demonstrated marked thinning of the inner retinal layers, consistent with chronic CRAO in the left eye. Given the rarity of CRAO in young individuals, a comprehensive systemic workup was performed, including hematological, inflammatory, coagulation, autoimmune, and cardiac evaluations. All investigations were within normal limits.

**Conclusion:**

CRAO can occur as a rare postoperative complication of cosmetic blepharoplasty. Early recognition, prompt management, and careful postoperative monitoring may prevent the risk of such complications.

## 1. Introduction

Central retinal artery occlusion (CRAO) is one of the few ophthalmic emergencies associated with sudden, severe, and sometimes irreversible vision loss. CRAO is becoming acknowledged as an ocular analog of ischemic stroke and a kind of acute retinal ischemia that needs immediate systemic assessment [1]. It is usually seen in the elderly with an annual incidence of about 1 per 100,000 individuals with underlying embolic or cardiovascular risk factors [2, 3]. The therapeutic window for CRAO is still short despite advancements in acute care, and therapy is frequently hampered by delayed presentation and a lack of widely accepted procedures [4]. Currently proposed interventions including ocular massage, intraocular pressure (IOP)–lowering therapy, and thrombolysis have limited efficacy and are highly time‐dependent, meaning that delayed presentation substantially diminishes the likelihood of visual recovery [1, 4]. The incidence of CRAO in young people is quite rare and warrants urgent investigation for atypical or iatrogenic causes [3]. Rare case reports have linked periocular and facial cosmetic procedures, including blepharoplasty, to these vascular ocular complications [5–7].

Blepharoplasty is usually considered a safe and routinely performed procedure; however, rare cases of postoperative visual loss have been documented [6, 7]. Recognizing uncommon but catastrophic ocular vascular problems has become more crucial due to the increase in cosmetic periocular surgeries worldwide. Proposed mechanisms include inadvertent intra‐arterial injection of local anesthetic or vasoconstrictive agents, reflex vasospasm, embolization, raised intraorbital pressure, or retrobulbar hemorrhage leading to compromised retinal perfusion [5–8]. These complications may occur even in young and healthy people with no identifiable systemic risk factors [9–11]. Although the majority of iatrogenic retinal vascular occlusions are described following dermal filler injections—largely because the high‐pressure injection technique can force filler material intra‐arterially, resulting in retrograde embolization through anastomotic vessels into the ophthalmic artery and subsequent obstruction of the central retinal artery [8]—blepharoplasty‐specific occlusions are extremely uncommon, especially in individuals who do not have obvious underlying risk factors.

The most often reported visual issues after blepharoplasty are orbital hemorrhage, optic neuropathy, and retinal vascular occlusions, while CRAO is still quite rare [11]. Due to the rarity of CRAO following blepharoplasty, the available literature is largely limited to isolated case reports and small case series, resulting in underrecognition of this blinding complication. Reporting such rare cases is important to improve understanding of possible mechanisms, enhance awareness among ophthalmologists, and emphasize preventive measures during periocular procedures [7, 8, 11]. We report a case of CRAO following cosmetic blepharoplasty in a young patient without systemic risk factors, highlighting potential mechanisms and the need for perioperative vigilance.

## 2. Case Presentation

**Figure 1:**
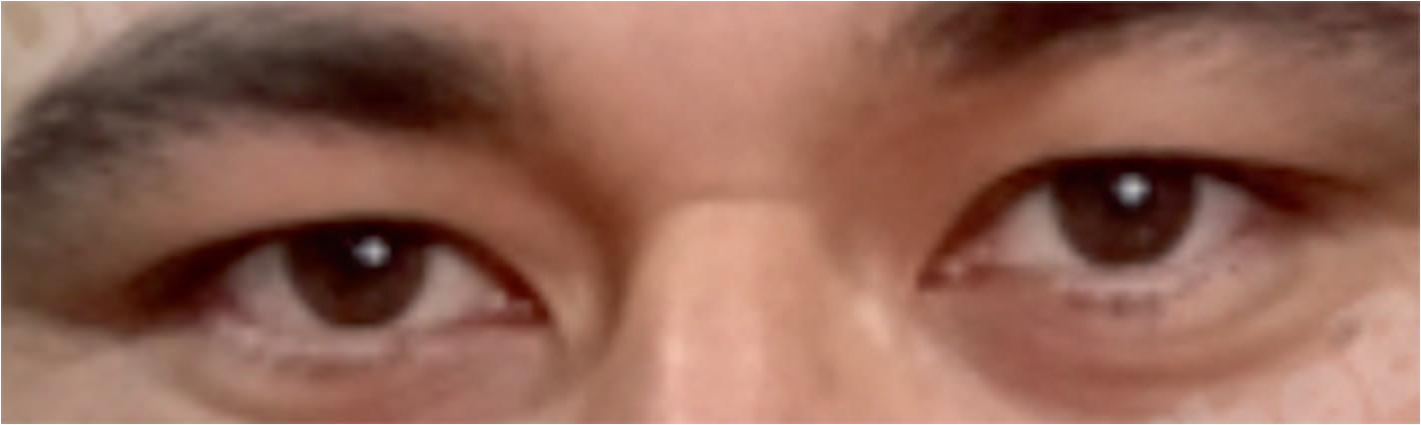
Showing excess periorbital fat around both eyes.

A 31‐year‐old man with no known systemic comorbidities presented to our clinic with a history of sudden, painless loss of vision in his left eye, occurring approximately 6 weeks earlier. Written informed consent was obtained from the patient for the publication of this case report and any accompanying images. Around the same time, the patient underwent routine bilateral cosmetic blepharoplasty for upper and lower lid dermatochalasis (Figure 1) in Afghanistan. According to the patient, the procedure involved bilateral upper and lower eyelid blepharoplasty performed under subdermal local anesthesia with 5 cc of lignocaine with adrenaline (1:200,000) in the upper and lower lids. The transconjunctival approach was used to access the excess periorbital fat. The total duration of the surgery was approximately 2 h. No intraoperative complications were reported, and the patient described the procedure as largely uneventful. Mild oozing from the incision sites was noted bilaterally after surgery, and he experienced a single episode of postoperative vomiting. At the end of the procedure, both eyes were padded with sterile strips and gauze and remained covered for 2 days. Upon removal of the eye pads, the patient noticed complete loss of vision in the left eye, whereas vision in the right eye remained normal.

**Figure 2:**
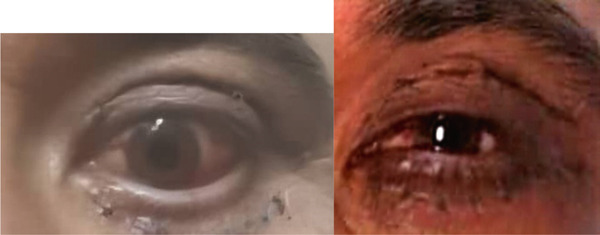
Clinical photographs of the left eye showing mid‐dilated pupil and nylon sutures in the upper and lower eyelids.

**Figure 3:**
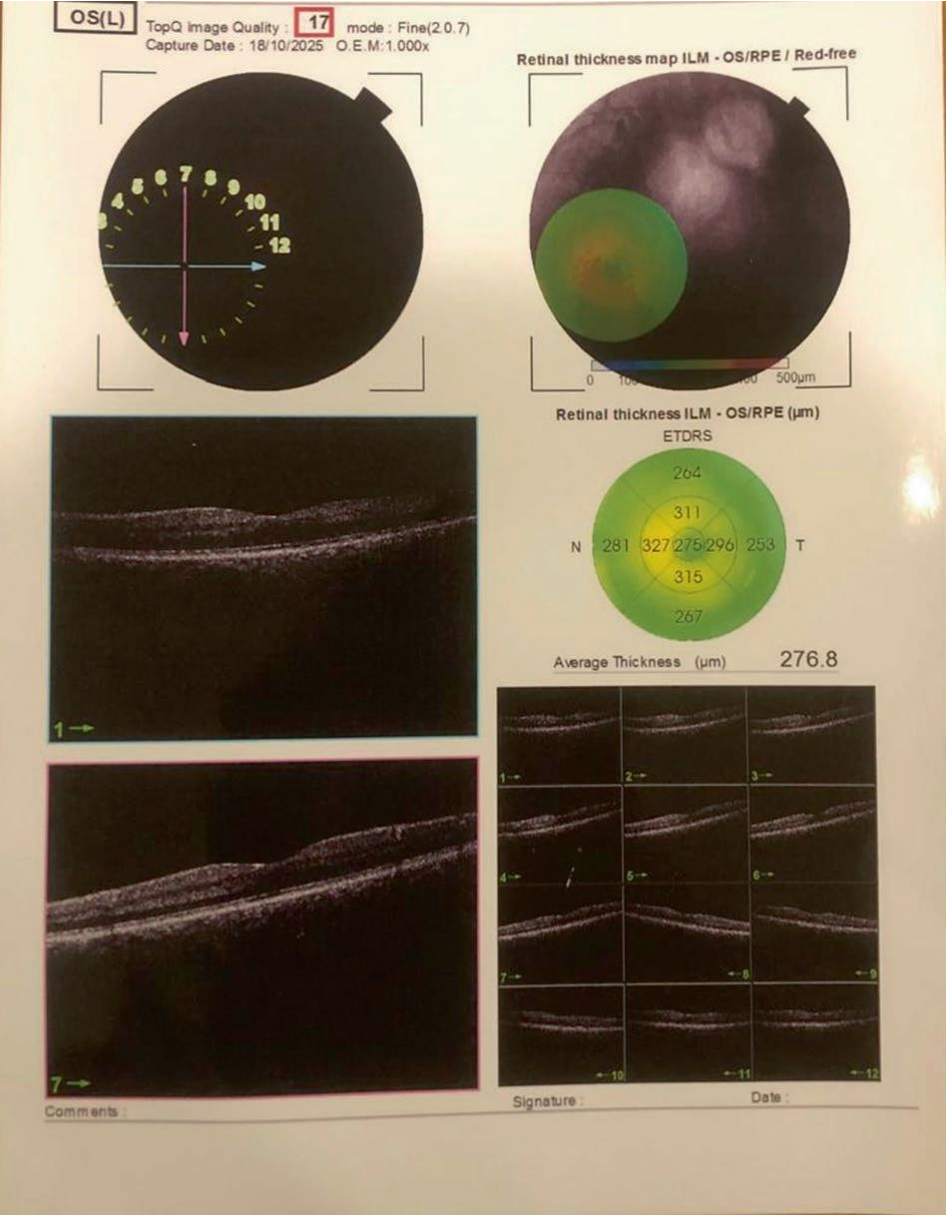
Spectral‐domain OCT of the left eye (acute phase) demonstrating hyperreflectivity and edema of the inner retinal layers.

Following the onset of visual loss, he presented to a local eye hospital for evaluation. Figure 2 demonstrates the mid‐dilated pupil and nylon sutures present in both the upper and lower eyelids. A spectral‐domain optical coherence tomography (OCT) scan was performed, which demonstrated hyperreflectivity and edema of the inner retinal layers, as illustrated in Figure 3. He was started on topical moxifloxacin, topical corticosteroids, and antiglaucoma eye drops. In view of the persistent visual deficit, the patient was subsequently referred to Pakistan for further assessment and management.

One and a half months after the primary procedure, he presented to our center. At presentation, the best corrected visual acuity (BCVA) was 20/25 in the right eye (OD), whereas the left eye (OS) had no perception of light (NPL). IOP was 14 mmHg in both eyes (OU). Pupillary examination revealed a round, regular, and reactive (RRR) pupil in the right eye. In contrast, the left pupil was mid‐dilated and nonreactive, with a positive relative afferent pupillary defect (RAPD). Slit‐lamp examination of the anterior segment was otherwise unremarkable, and posterior segment evaluation did not reveal additional abnormalities in both eyes.

**Figure 4:**
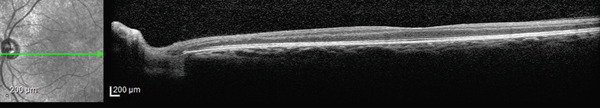
OCT of the left eye at 6‐week follow‐up demonstrating marked thinning of the inner retinal layers consistent with chronic CRAO.

**Figure 5:**
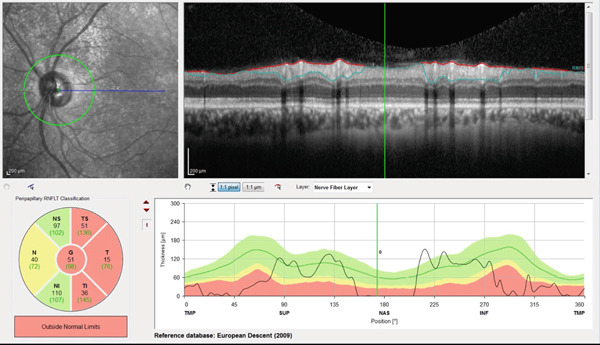
OCT of the left eye (additional cross‐section) confirming inner retinal atrophy.

OCT performed at our institution demonstrated marked thinning of the inner retinal layers, as shown in Figures 4 and 5, findings consistent with chronic CRAO in the left eye. Fluorescein angiography and fundus photography were not performed at the original treatment facility. The diagnosis of chronic CRAO was based on the clinical history of abrupt, painless monocular visual loss following periocular surgery, a positive RAPD, and OCT evidence of significant inner retinal layer thinning.

Since CRAO is rare in young individuals, a comprehensive systemic workup was undertaken to identify potential predisposing factors. The patient had no known history of hypertension, diabetes mellitus, cardiovascular disease, or thromboembolic disorders, and the fellow eye remained completely asymptomatic following surgery. Investigations included complete blood count (CBC), erythrocyte sedimentation rate (ESR), C‐reactive protein (CRP), serum homocysteine levels, HbA1c, proteins C and S levels, factor V Leiden mutation analysis, prothrombin levels, renal and liver function tests, and thyroid function tests. An autoimmune evaluation, including antinuclear antibody (ANA), antimitochondrial antibody (AMA), antismooth muscle antibody (ASMA), antigastric parietal cell antibody (AGPCA), and both cytoplasmic and perinuclear antineutrophil cytoplasmic antibodies (c‐ANCA and p‐ANCA), antiphospholipid antibodies (both serum antiphospholipid IgG and IgM), lupus anticoagulant, and anticardiolipin (both IgG and IgM) antibodies, was also performed. Additionally, cardiac echocardiography was obtained to exclude a cardioembolic source. Both MRI and CT scans of the brain and orbits with contrast also reported normal findings. All investigations were within normal limits.

In the absence of identifiable systemic or hematologic abnormalities, the precise cause of the retinal arterial occlusion remained uncertain. Given the temporal association with the recent eyelid surgery, the patient was ultimately diagnosed with unilateral idiopathic CRAO occurring after cosmetic blepharoplasty.

## 3. Discussion

This case describes unilateral CRAO following bilateral cosmetic blepharoplasty in a 31‐year‐old man with no identifiable systemic risk factors, resulting in permanent loss of light perception. CRAO typically affects patients in the sixth to seventh decades with atherosclerotic cardiovascular disease, with an annual incidence of approximately 1–2 per 100,000 [12]. When CRAO occurs in younger patients (≤ 45 years), the etiological spectrum shifts to include hypercoagulable states, vasculitis, structural cardiac anomalies, patent foramen ovale, and iatrogenic causes, though a substantial proportion remains unexplained despite thorough investigation [9].

Several pathways may explain the mechanisms for retinal vascular occlusion following periocular surgery. The most widely cited is inadvertent intra‐arterial injection of local anesthetic containing a vasoconstrictor. The rich anastomotic periocular arterial network, where terminal branches of the ophthalmic artery interconnect with facial and superficial temporal arteries, means that even small intra‐arterial volumes of adrenaline‐containing anesthetic can propagate retrograde toward the central retinal artery or induce intense vasospasm [12, 13]. A second mechanism is raised intraorbital pressure from perioperative hemorrhage or edema, potentially exacerbated by prolonged bilateral eye padding [14]. In this patient, mild bilateral oozing and postoperative vomiting (which transiently raises intraorbital venous pressure) may have compounded perfusion compromise in an already vulnerable vascular bed. A third possibility is direct mechanical compression of the globe during prolonged surgical retraction.

The unilaterality of the complication argues against a bilateral systemic cause such as hemodynamic collapse or central embolic shower, instead localizing the mechanism to factors specific to the operative field of the left eye, which is a pattern consistent with prior reports of asymmetric injection volumes or technique variation between sides [15].

The existing literature on blepharoplasty‐associated visual loss provides useful context for the present case. Kelly and May [14] reported a case of CRAO after cosmetic blepharoplasty in a 70‐year‐old man who developed a right orbital hemorrhage after bilateral lower lid blepharoplasty with fat removal; emergency decompression restored vision to 20/20. In contrast, our patient presented 6 weeks after surgery, well beyond any therapeutic window, and bilateral occlusive padding may have masked the onset of visual loss during the period when intervention might still have been beneficial. Mahaffey and Wallace [7], in their review of more than 55 cases of postblepharoplasty blindness, identified orbital hemorrhage as the most frequent mechanism, while also noting cases in which vasoconstrictive local anesthetics appeared contributory, a mechanism directly relevant to the lignocaine‐adrenaline technique used here. Van Der Kelen and Mommaerts [6] described visual loss after blepharoplasty performed under epinephrine‐containing local anesthesia, with diagnoses including acute angle‐closure glaucoma and posterior ischemic optic neuropathy rather than CRAO per se; nevertheless, their cases similarly support the possibility of epinephrine‐mediated vascular compromise. Across these reports, outcomes ranged from complete recovery to permanent blindness, with speed of recognition and intervention appearing to determine prognosis; our case reinforces that pattern.

The comprehensive systemic evaluation at our center, which comprised hematological, thrombophilic, autoimmune, metabolic, and cardiac workup, was entirely unremarkable. This is consistent with the emerging literature: In a recent retrospective study of young patients with retinal artery occlusion without typical cardiovascular risk factors, vasculitis and patent foramen ovale were the most frequently identified causes, but a substantial proportion remained idiopathic despite thorough workup [9]. The negative investigation profile in our patient is consistent with—though does not confirm—an iatrogenic mechanism.

A limitation of this case is the absence of fundus photography and fluorescein angiography, which would have provided more objective evidence of retinal artery nonperfusion during the acute stage. These studies were not available at the initial treatment facility, and by the time the patient presented to our center approximately 6 weeks after symptom onset, the acute retinal changes had already evolved into chronic inner retinal atrophy. However, the evolution of the OCT findings provided objective diagnostic confirmation. The initial OCT demonstrated hyperreflectivity and edema of the inner retinal layers—consistent with acute cytotoxic ischemia of the ganglion cell and inner plexiform layers supplied exclusively by the central retinal artery [16]. By 6 weeks, the OCT had evolved to show marked inner retinal atrophy, reflecting irreversible neuronal loss characteristic of chronic CRAO.

No acute interventional therapy for CRAO was administered at the initial facility. This reflects a recognized challenge: There is no guideline‐endorsed treatment with proven efficacy, and the therapeutic window is extremely narrow [17]. Conservative interventions such as ocular massage, paracentesis, and vasodilators have shown no clear benefit [18]. Limited evidence suggests intravenous thrombolysis with alteplase or tenecteplase within 4.5 h offers the strongest signal for visual recovery, though large, randomized trials are lacking [18, 19]. Intra‐arterial thrombolysis may extend the treatment window, but results remain inconsistent, and the EAGLE trial demonstrated no superiority of intra‐arterial tPA over conservative standard treatment in acute CRAO [20]. By the time this patient reached our center, the inner retinal atrophy made any intervention futile. The outcome: NPL is consistent with the natural history of complete, untreated CRAO, where spontaneous meaningful visual recovery occurs in fewer than one‐third of patients.

Other diagnoses, such as ophthalmic artery blockage, posterior ischemic optic neuropathy, compressive optic neuropathy due to orbital hemorrhage or elevated orbital pressure, and traumatic optic neuropathy, were taken into consideration due to the severe visual outcome of no sense of light. However, persistent CRAO was the most likely diagnosis based on the OCT pattern of inner retinal atrophy, the presence of RAPD, normal neuroimaging, and a negative systemic workup.

From a prevention standpoint, this case carries several practical implications. Surgeons performing periocular procedures should employ careful aspiration techniques prior to local anesthetic infiltration to minimize intra‐arterial injection risk [12, 13]. Postoperative bilateral occlusive padding should be applied judiciously, as it prevents the patient from detecting acute visual loss during the narrow therapeutic window. Where facilities allow, a brief visual acuity check should be performed before discharge from the operating suite.

In conclusion, this case illustrates that CRAO can occur in close temporal association with cosmetic blepharoplasty, with potentially devastating consequences, capable of occurring in young, systemically healthy individuals. The mechanism is likely multifactorial: perioperative vasoconstrictor injection, raised intraorbital pressure, and postoperative edema may all contribute. The unilaterality of the event, its temporal relationship to surgery, and the absence of systemic risk factors are collectively suggestive of an iatrogenic mechanism, although a definitive causal link cannot be established from a single case. Heightened awareness among periocular surgeons and early ophthalmological assessment of any postoperative visual complaint are essential for preventing this irreversible complication.

## Funding

No funding was received for this manuscript.

## Consent

Written informed consent was obtained from the patient for publication of this case report and accompanying images. Ethical review committee (ERC) approval was obtained from the Aga Khan University. (ERC Approval Number: 2026‐12557‐39575).

## Conflicts of Interest

The authors declare no conflicts of interest.

## Supporting information


**Supporting Information** Additional supporting information can be found online in the Supporting Information section. 

## Data Availability

The data that support the findings of this study are available on request from the corresponding author. The data are not publicly available due to privacy or ethical restrictions.
