# Supplementary material for: Central Retinal Artery Occlusion Following Cosmetic Blepharoplasty in a Young Patient: A Case Report
Source: Case Rep Ophthalmol Med. 2026 Jul 22;2026:5972407. doi: 10.1155/crop/5972407 (PMC13392415; doi:10.1155/crop/5972407)
Supplement: Supplementary file 1 — Supporting Information Additional supporting information can be found online in the Supporting Information section. [file CROP-2026-5972407-s001.doc]

# Consent Form for Case Reports

Case Report: Central retinal artery occlusion after blepharoplasty in a young man

**Principal Investigator:**

Dr. Haroon Tayyab,

*MBBS(AKU), FCPS(Opth), FCPS(Vitreoretina)*

*FRCS(Glasg), FRCSEd, FACS, Fellowship in Retina (Japan)*

Interim Chair Clinical Services / Associate Professor

The Aga Khan University
Stadium Road, P.O. Box 3500, Karachi 74800, Pakistan

Email: haroon.tayyab@aku.edu

You are being asked to consider allowing Dr. Haroon Tayyab to use information about your Central retinal artery occlusion after blepharoplasty to write what is called a case report. Case reports are typically used to share new unique information experienced by one patient during his/her clinical care that may be useful for other physicians and members of a health care team. A case report may be published in print and/or via internet dissemination for others to read, and/or presented at a conference. This form explains the purpose of this case report. Please read this form carefully and take your time to make your decision and ask any questions that you may have.

The purpose of this case report is to inform other physicians that Central retinal artery occlusion may be a rare occurrence after blepharoplasty

Your information being used for this case report includes your age, gender, symptoms you experienced, and the treatment given to you.

Dr. Haroon Tayyab is obligated to protect your privacy and not disclose your personal information (information about you and your health that identifies you as an individual e.g. name, date of birth, medical record number). When the case report is published or presented, your identity will not be disclosed.

Although your personal information collected or obtained will be kept confidential and protected to the fullest extent of the law, there is a limited risk associated with this case report that could result in a loss of confidentiality by virtue of your unique experience.

You will not directly benefit from participating in this case report. The information that can be shared with other health care professionals, however, may improve the care that is received by others in the future.

Allowing your information to be used in this report will not involve any additional costs to you. You will not receive any compensation.

Taking part in this case report is your choice (voluntary). You may choose not to take part, or you may change your mind at any time. However, once the case report is written and published, it will not be possible for you to withdraw it. Your decision will not result in any penalty or loss of benefits to which you are entitled, including the quality of care you receive.

You will be told about any new information relating to this case report that may affect you.

Your signature below means that you have read the above information about this Case Report and have had a chance to ask questions to help you understand how your information will be used and that you give permission to allow your information to be used in this case report.

If you have any questions, please contact Dr. Haroon Tayyab at [haroon.tayyab@aku.edu](mailto:haroon.tayyab@aku.edu)

**SUBJECT CONSENT TO PARTICIPATE**

Case Report Title: Central retinal artery occlusion after blepharoplasty in a young man

Name of Participant: Syed Mohammad Bakir

Participant/Substitute decision-maker

By signing this form, I confirm that:

- The case report has been fully explained to me, and all of my questions have been answered to my satisfaction
- I have been informed of the risks and benefits, if any, of allowing my information to be used in this case report
- I have read each page of this form
-
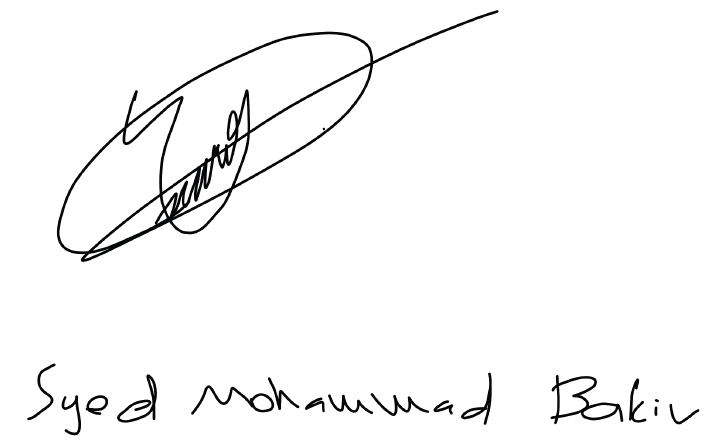
I authorize access to my personal health information (medical record) as explained in this form
- I have agreed to participate in this case report

Syed Mohammad Bakir December 31^st^ ,2025

Name of Participant/Relationship Signature Date

of Substitute Decision-maker (print)
